# Effect of Functional Group on the Catalytic Activity of Lipase B from *Candida antarctica* Immobilized in a Silica-Reinforced Pluronic F127/α-Cyclodextrin Hydrogel

**DOI:** 10.3390/gels8010003

**Published:** 2021-12-21

**Authors:** Cédric Decarpigny, Anne Ponchel, Eric Monflier, Rudina Bleta

**Affiliations:** University of Artois, CNRS, Centrale Lille, ENSCL, Univ. Lille, UMR 8181-UCCS-Unité de Catalyse et Chimie du Solide, F-62300 Lens, France; cedric.decarpigny@univ-artois.fr (C.D.); anne.ponchel@univ-artois.fr (A.P.); eric.monflier@univ-artois.fr (E.M.)

**Keywords:** CALB, supramolecular hydrogel, silica, surface functionalization, α-cyclodextrin, Pluronic F127, 2,5-furandicarboxylic acid

## Abstract

Surface modification plays a key role in the fabrication of highly active and stable enzymatic nanoreactors. In this study, we report for the first time the effect of various functional groups (epoxy, amine, trimethyl, and hexadecyl) on the catalytic performance of lipase B from *Candida antarctica* (CALB) incorporated within a monolithic supramolecular hydrogel with multiscale pore architecture. The supramolecular hydrogel formed by host-guest interactions between α-cyclodextrin (α-CD) and Pluronic F127 was first silicified to provide a hierarchically porous material whose surface was further modified with different organosilanes permitting both covalent anchoring and interfacial activation of CALB. The catalytic activity of nanoreactors was evaluated in the liquid phase cascade oxidation of 2,5-diformylfuran (DFF) to 2,5-furandicarboxylic acid (FDCA) under mild conditions. Results showed that high FDCA yields and high efficiency conversion of DFF could be correlated with the ability of epoxy and amine moieties to keep CALB attached to the carrier, while the trimethyl and hexadecyl groups could provide a suitable hydrophobic-hydrophilic interface for the interfacial activation of lipase. Cationic cross-linked β-CD was also evaluated as an enzyme-stabilizing agent and was found to provide beneficial effects in the operational stability of the biocatalyst. These supramolecular silicified hydrogel monoliths with hierarchical porosity may be used as promising nanoreactors to provide easier enzyme recovery in other biocatalytic continuous flow processes.

## 1. Introduction

In the current context where plastic pollution is becoming a source of increasing concern and a pressing environmental issue, the development of new ecofriendly catalytic processes for the conversion of renewable biomass into high value biodegradable materials represents a major challenge for sustainable development [1,2,3,4,5]. In particular, bioplastics are currently experiencing a strong development with a global production of 2.11 million tons in 2020, which is set to increase to approximately 2.87 million tons in 2025 [6].

2,5-Furandicarboxylic acid (FDCA), obtained from 5-hydroxymethylfurfural (HMF) oxidation, is a key building block for the production of bio-based polymers, such as polyamides and polyesters, and has recently been identified as a potential substitute of petroleum-based terephthalic acid (TPA), the main building block of polyethylene terephthalate (PET) used in food packaging and plastic bottles [7]. Polyethylene furanoate (PEF) produced by polycondensation of ethylene glycol and FDCA, has demonstrated superior barrier and thermal properties with respect to PET, and is expected to enter the market very soon, in the early 2023 [6].

To date, various metal-based catalysts such as gold, platinum, and palladium nanoparticles, as well as mixed oxides, have been used as heterogeneous catalysts for the production of FDCA [8]. However, those catalysts require high temperatures and pressures, as well as strongly alkaline conditions that tend to produce large amounts of salts, thus limiting the development of the process on a large scale [9].

To help meet growing demands for green and sustainable chemicals, biocatalysis has emerged as a promising technology over the past twenty years [10,11,12]. Enzymes are particularly suitable biocatalysts for biomass conversion as they operate under eco-friendly conditions (moderate temperature, atmospheric pressure, and physiological pH) and exhibit excellent selectivity (regio-, chemo-, and enantio-), thus reducing the formation of by-products [13,14,15,16]. Nevertheless, despite the recent progress in biomolecular engineering, the industrial applications of enzymes have been hampered by a poor long-term stability and difficulties in recycling the biocatalyst. To overcome those limitations, immobilization of enzymes on a solid carrier is a promising technology to increase their operational stability for reuse in several cycles, thus reducing the biocatalyst cost [10].

Hydrogels are three-dimensional macromolecular networks composed of hydrophilic polymers interconnected trough physical or chemical nodes [17]. The macromolecular network can incorporate large amounts of water, generally more than 80% of the total mass, conferring to the hydrogel biocompatibility properties [18]. Unlike synthetic hydrogels, supramolecular hydrogels are reversible in nature because the crosslinking nodes are formed by non-covalent bonds [19]. The first cyclodextrin (CD)-based supramolecular hydrogels reported in the literature are those formed between poly (ethylene glycol) (PEG) and α-CD [20]. Threading of several α-CDs along the chain of a high molecular weight PEG (>10,000 Da) was shown to cause the formation of insoluble poly*pseudo*rotaxanes that tended to self-assemble into three-dimensional organized supramolecular nanostructures [21].

In the case of nonionic Pluronic PEO-PPO-PEO triblock copolymers, where thinner PEO blocks flank the middle PPO block, it has been shown that β-CD slides along the hydrophilic PEO blocks to selectively thread the middle hydrophobic PPO blocks, while α-CD selectively threads the flanking PPO blocks forming poly*pseudo*rotaxanes in high yields [22]. As demonstrated by Travelet et al. [23,24], the growth of columnar nanocrystallites resulting from the self-organization of several poly*pseudo*rotaxanes is believed to play a key role in maintaining the hydrogel in a stable water-swollen state.

The ability of hydrogels to incorporate high levels of proteins, cells, antibodies, peptides, and genes has been widely described in the literature, especially for biomedical applications [25,26,27]. Importantly, the interconnected three-dimensional structure of supramolecular PEG/α-CDs hydrogels [28] can provide a favorable microenvironment for the incorporation of biomolecules, in particular enzymes [29]. However, under acidic conditions [30], or in the presence of molecules comprising acidic functions, such as the FDCA, these hydrogels suffer from lack of stability due to the unthreading of α-CD from the polymer chain, thus resulting in partial dissolution of the interconnected 3D structure and further release of the biocatalyst. To overcome those limitations, solidification of the supramolecular hydrogel network through silicification is a versatile strategy for enhancing the biocatalyst stability and improving its catalytic performance. Indeed, surface silanols can be further functionalized with various chemically reactive and hydrophobic groups, permitting effective covalent anchoring and interfacial activation of lipases. Nevertheless, in the specific case of α-CD-based hydrogels, the intercalation of silica layers with the poly*pseudo*rotaxanes, as well as the presence of a thick hydration shell on the hydrogel surface, may hinder the accessibility of surface silanols to the silylating agent, thus making surface functionalization challenging.

To date, the functionalization of silicified hydrogels has not been thoroughly investigated and little work has been done on their use as host matrices for the immobilization of enzymes for catalytic applications [29,31]. However, these hybrid systems could provide an interesting alternative to mesoporous silicas, such as MCM-41 [32] or SBA-15 [33], owing to their tunable surface properties, hierarchical pore structure, and promising mechanical properties [34,35].

Herein, we present a new approach that combines the concepts of supramolecular chemistry and sol-gel process, together with surface functionalization, to develop hierarchically porous monoliths able to incorporate high amounts of *Candida antarctica* lipase B (CALB). CALB has previously been identified as one of the most important lipases in industrial applications owing to its high selectivity toward secondary amines and secondary alcohols [36,37], as well as its high resistance toward H_2_O_2_ [38,39]. Prior to CALB immobilization, we have investigated the effect of a wide variety of organosilanes on the surface properties of the silicified hydrogel. To highlight the advantages and limitations of different functional groups, the catalytic performance of nanoreactors was evaluated in the liquid phase oxidation of 2,5-diformylfuran (DFF) to FDCA. In addition to the effect of reactive and hydrophobic groups, the impact of cationic cross-linked β-CD as an enzyme-stabilizing agent was also examined.

## 2. Results

### 2.1. Silicification of the Pluronic F127/α-CD Hydrogel

**Figure 1:**
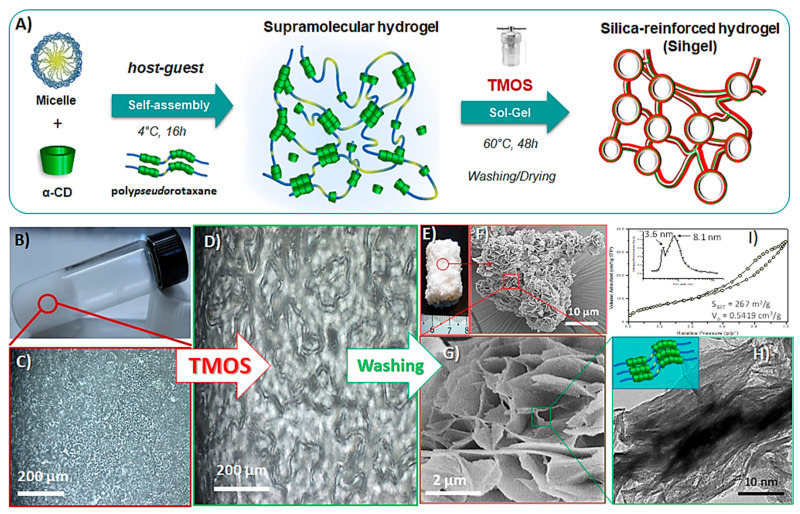
Schematic illustration of the procedure used for the preparation of silica-reinforced Pluronic F127/αCD hydrogel (**A**). Visual aspect of the supramolecular hydrogel (**B**). Polarized optical microscopy images of the hydrogel before (**C**) and after silicification (**D**) showing birefringent textures. Visual aspect of the porous monolith after annealing at 60 °C and removal of the excess of template (**E**). SEM (**F**,**G**) and TEM (**H**) micrographs of the silicified hydrogel and corresponding N_2_ adsorption results (**I**).

A hierarchically porous hybrid material was prepared using the Pluronic F127/α-CD hydrogel as template and tetramethoxysilane (TMOS) as silica source (Figure 1A) [31]. First, host-guest interactions between poly (ethylene oxide) (PEO) blocks of F127 and α-CD in aqueous phase led to total dissociation of the micelles. Then, the self-assembly of poly*pseudo*rotaxanes resulted in a physically cross-linked hydrogel and its further silicification through hydrolysis and condensation of TMOS yielded a three-dimensional silica-reinforced network containing a thin layer of hydrogel. Pristine hydrogel displayed a thixotropic behavior forming a stable gel structure at rest (Figure 1B), but becoming fluid when agitated. Moreover, the thixotropic degree became more pronounced with increasing the polymer loading (Figure S1, ESI). Optical microscopy images in polarized light showed weak birefringence textures developed by nanocrystallites within pristine hydrogel (Figure 1C), while addition of TMOS greatly accelerated their growth (Figure 1D). After annealing at 60 °C for 48 h, followed by excess template removal, the mixture solidified into a porous monolith (Figure 1E). SEM images indicated formation of structures with flower-like patterns (Figure 1F) made-up of petals with 40–50 nm thickness (Figure 1G), while TEM micrographs revealed that the pore wall was comprised of particles with fibber-like morphology, consistent with the elongated cylindrical structure of poly*pseudo*rotaxanes (Figure 1H). The surface area and pore volume determined by N_2_-adsorption analysis (Figure 1I) were 267 m^2^/g and 0.542 cm^3^/g respectively, while mesopores had average 3.6 nm and 8.1 nm diameters originating from individual poly*pseudo*rotaxanes and channel-like nanocrystallites respectively (Figure 1I, inset).

### 2.2. Effect of Functionnal Groups

#### 2.2.1. Method for Surface Functionalization and Immobilization of CALB

**Figure 2:**
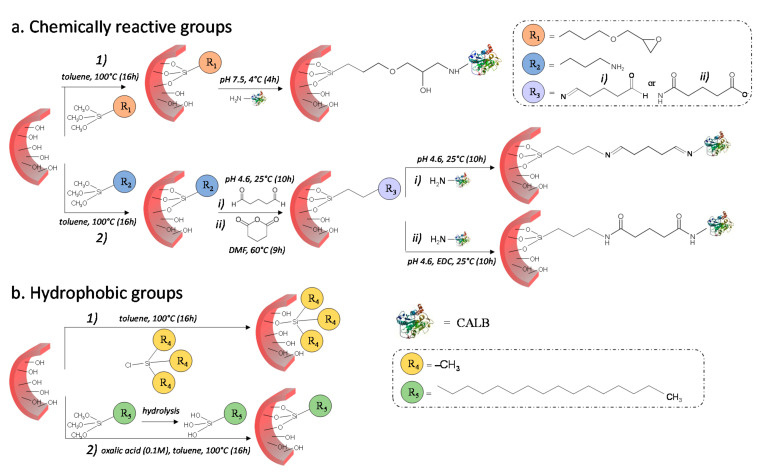
Schematic illustration of the procedure used for the preparation of bifunctional nanoreactors. (**a**) Enzyme immobilization on the silicified hydrogel functionalized with chemically reactive groups necessary for the covalent binding of the lipase. (**a1**) The epoxy function alkylates the primary amine of the enzyme yielding a secondary amine. (**a2**) The amine group in the silica wall reacts with the primary amine of lipase through a bifunctional spacer providing two imine (GAH) or two amide (GAC) bonds. (**b**) Functionalization of pore wall with trimethylsilyl (**b1**) and hexadecyl (**b2**) groups necessary for the interfacial activation of the lipase.

Prior to enzyme immobilization, the surface of silica-reinforced hydrogel was modified with different functional groups from four organosilanes (Figure 2). (3-glycidoxypropyltrimethoxysilane (GPTMS) and 3-aminopropyl trimethoxysilane (APTMS) were used as chemically reactive groups for the covalent binding of CALB, while chlorotrimethylsilane (CTMS) and hexadecyltrimethoxysilane (HDTMS) were employed as hydrophobic functions for its interfacial activation.

Surface modification with GPTMS (Figure 2(a1)) produces a terminal epoxy group which further reacts with the nucleophilic primary amines of lysine residues on CALB surface through a ring opening reaction, yielding a secondary amine [40]. Owing to its high reactivity, the epoxy group can also react with other nucleophiles, such as the hydroxy groups available on the hydrogel surface and the thiol moieties present on the cysteine residues of the enzyme. As the ring opening reaction with primary amines is usually reported to be more favorable under neutral or moderate alkaline conditions [41], grafting of CALB was carried out in phosphate buffer at pH 7.5.

Conversely, the rate at which the imine bond forms has been reported to be greatest near a pH of 5.0 [42]. Therefore, after surface functionalization with aminopropyl groups from APTMS, the reaction with the bifunctional spacer, i.e., the glutaraldehyde (GAH) or the glutaric anhydride (GAC) was carried out in acetate buffer (pH 4.6) (Figure 2(a2)). GAH provides an aldehyde group that reacts with the primary amine of lysine providing an imine bond (Schiff base) (Figure 2(a2i)), while GAC gives access to the carboxyl group, which subsequently reacts with amino groups of lysine to form an amide (Figure 2(a2ii)).

In the process of hydrophobization of silicified hydrogel (Figure 2b), CTMS first reacts with surface silanols yielding trimethylsilyl groups (Si(CH_3_)_3_) (Figure 2(b1)) and releasing hydrochloric acid (HCl). On the other hand, in the case of HDTMS, because of the high degree of hydrophobicity of the hexadecyl group (C16), the silane was first hydrolyzed in an oxalic acid solution (0.1 M), then grafted through condensation with surface silanols (Figure 2(b2)).

To examine the impact of the different functional groups on the catalytic activity of CALB, three series of bifunctional materials were prepared by simultaneous functionalization with (i) CTMS and GPTMS, (ii) HDTMS and APTMS activated with GAH, and (iii) CTMS and APTMS using GAH or GAC as bifunctional spacers. The resulting materials were characterized using TG analysis together with ATR-FTIR spectroscopy.

#### 2.2.2. Characterization of Biocatalysts

(a) Sihgel@CTMS@GPTMS@CALB

**Figure 3:**
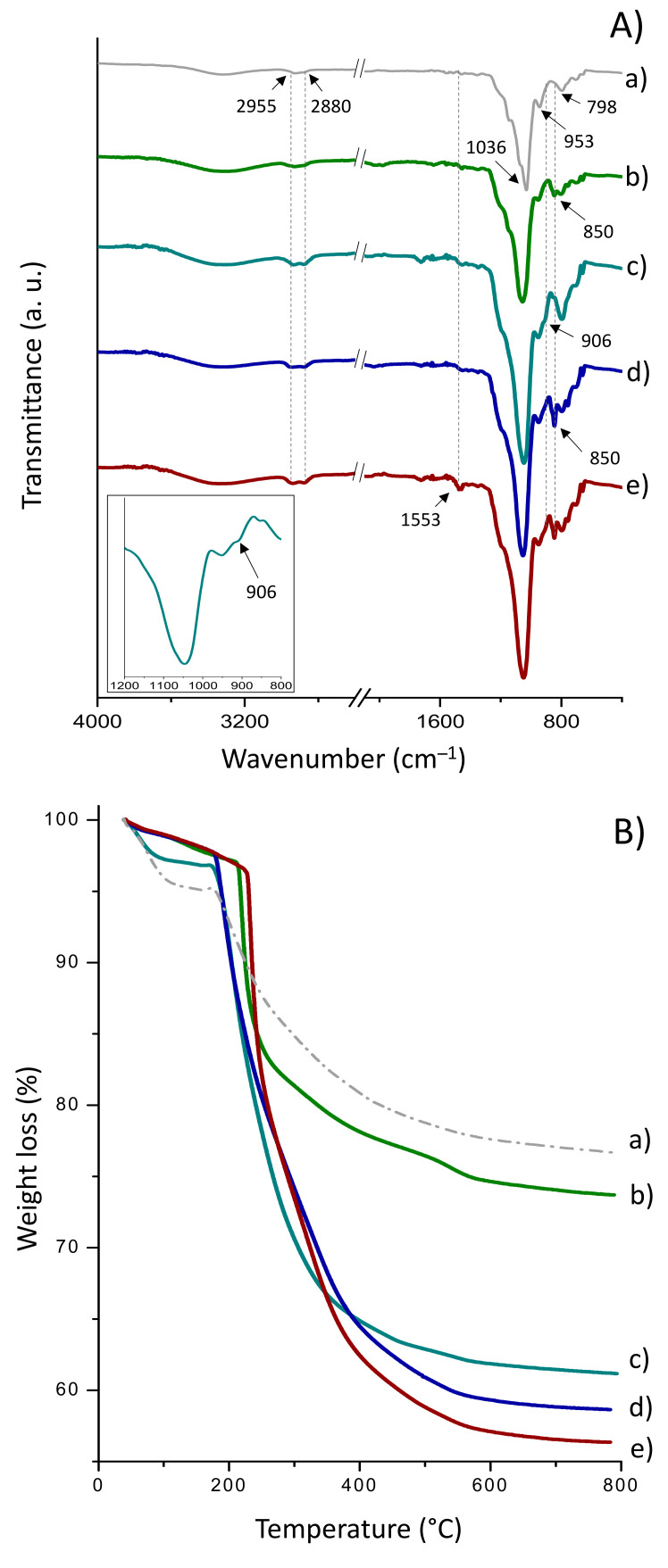
ATR FTIR spectra (**A**) and TG curves (**B**) for (**a**) as-synthesized Sihgel, (**b**) Sihgel@CTMS, (**c**) Sihgel@GPTMS, (**d**) Sihgel@CTMS@GPTMS, and (**e**) Sihgel@CTMS@GPTMS@CALB. The low intensity of the band at 906 cm^−1^ suggests that part of epoxy groups may have been opened into diols during reaction with surface silanols.

The FT-IR spectra of CTMS- and GPTMS-functionalized Sihgel, before and after CALB immobilization, are shown in Figure 3A. The as-synthesized silicified hydrogel (Figure 3(Aa)) displays a strong absorption band at 1036 cm^−1^ ascribed to the asymmetric stretching vibrations of the Si–O–Si bond [43] that overlaps with the ring vibrational modes of glycosyl units in α-CD [44]. Two additional bands appear at 798 cm^−1^ and 953 cm^−1^ arising respectively from the symmetric stretching vibrations of Si–O–Si bond and Si–OH groups [43]. Furthermore, the bands in the 2960–2890 cm^−1^ region are typical of C–H vibration from both α-CD and Pluronic F127.

Grafting of trimethylsilyl groups on the Sihgel@CTMS material (Figure 3(Ab)) was confirmed by the increase in intensity of the symmetric and asymmetric C–H stretching vibrations from aliphatic –CH_3_ groups at 2880 cm^−1^ and 2955 cm^−1^ respectively, as well as the Si–C stretching vibration at 850 cm^−1^ from –O–SiCH_3_ end groups [43,45]. Upon surface modification with GPTMS (Figure 3(Ac)), a new band appeared at 906 cm^−1^ originating from the epoxide ring vibration [46]. However, its low intensity suggests that part of epoxides may have been opened into diols during reaction with surface silanols. On the other hand, in the case of Sihgel@CTMS@GPTMS (Figure 3(Ad)), the bands at 850 cm^−1^ and 906 cm^−1^ confirm surface functionalization with both hydrophobic and reactive groups. Moreover, upon enzyme immobilization (Figure 3(Ae)), the high intensity vibration at 1553 cm^−1^ originating from the N–H bending of free amines in protein is consistent with successful anchoring of the lipase.

The loading amount of organic moieties was determined by TG analysis in the temperature range between 180 °C and 800 °C (Figure 3B). The weight loss below 180 °C was not taken into consideration as it belongs to loss of solvent physically adsorbed into the pores. From the difference between the weight loss of the silicified hydrogel and the weight loss of bare sol-gel silica, the amount of incorporated hydrogel was found to be 13.8%. In the typical curves of CTMS- (Figure 3(Bb)) and GPTMS-functionalized Sihgel (Figure 3(Bc)), the sharp mass drops above 180 °C arise from the decomposition of organic trimethyl (4.5%) and glycidiloxypropyl (17.4%) moieties respectively (Table 1). Importantly, simultaneous functionalization with CTMS and GPTMS resulted in a total weight loss of 20.7%, which was close to that recorded on Sihgel@CTMS and Sihgel@GPTMS taken together (21.9%) (Table 1).

The grafting densities (Dg) on Sihgel@CTMS and Sihgel@GPTMS were found equal to be 45 mg/g and 174 mg/g respectively. Moreover, using Equation (1) (see Experimental part), the corresponding surface coverages (S_cov_) were determined to be 0.24 chain/nm^2^ and 0.41 chain/nm^2^ respectively. The maximum grafting densities (D_g_^max^) of trimethyl and glycidoxypropyl groups that could theoretically be grafted on the surface of the host matrix were also calculated assuming that GPTMS was fully hydrolyzed and that trimethyl groups from CTMS formed a monolayer on the hydrogel surface. Therefore, considering that trimethyl moieties occupy a surface area of 0.43 nm^2^ [47] and that the surface occupied by glycidoxypropyl groups is 0.2 nm^2^ [48], the maximum grafting densities were determined to be 57 mg/g and 105 mg/g respectively (Table 1). By comparing the experimental data with the theoretical predictions, it appears that CTMS most likely forms a thin monolayer on the hydrogel surface, whereas GPTMS gives rise to a thicker layer, probably due to the higher reactivity and sol-gel reticulation of GPTMS network [49]. Furthermore, the approximate amount of grafted protein, determined by the difference in the mass loss between Sihgel@CAG@CALB (B,e) and Sihgel@CAG (B,d), was determined to be 2.5% (Table 1).

(b) Sihgel@HDTMS@APTMS@CALB

**Figure 4:**
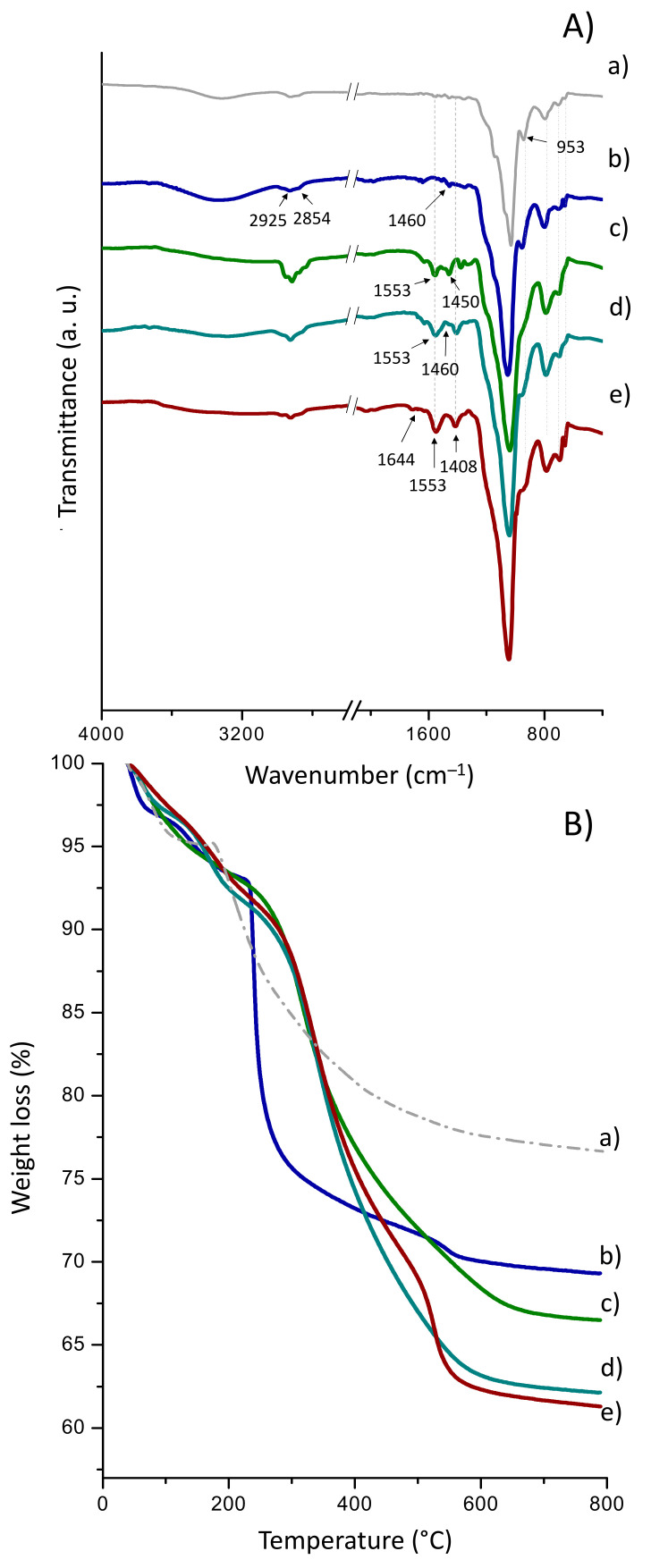
ATR FTIR spectra (**A**) and TG curves (**B**) for (**a**) as-synthesized Sihgel, (**b**) Sihgel@HDTMS, (**c**) Sihgel@APTMS, (**d**) Sihgel@HDTMS@APTMS-GAH, and (**e**) Sihgel@HDTMS@APTMS-GAH@CALB.

Figure 4A displays the FTIR spectra of HDTMS- and APTMS-functionalized material before and after enzyme immobilization. Relative to pristine Sihgel (Figure 4 (Aa)), the spectrum of HDTMS-functionalized Sihgel (Figure 4 (Ab)) displays a new vibration band at 1460 cm^−1^ characteristic of the –CH_2_ and –CH_3_ bendings in the hexadecyl chains [43]. Moreover, the bands at 2925 and 2854 cm^−1^ assigned respectively to the asymmetric and the symmetric stretching vibrations of CH_2_ groups [50] further confirm grafting of the hydrophobic silane. On the other hand, the spectrum of APTMS-functionalized Sihgel (Figure 4 (Ac)) presents two vibrations at 1553 cm^−1^ and 1454 cm^−1^ consistent with the N–H bending of the primary amine and the symmetric CH_2_ bending of Si–CH_2_ groups respectively. Further, the bifunctional material (Sihgel@HDTMS@APTMS-GAH) displayed the typical vibration bands of both silanes indicating successful surface modification (Figure 4 (Ad)). Notably, upon enzyme immobilization, the increase in intensity of the vibration at 1553 cm^−1^ corresponding to N–H bending of free amines in protein, together with the band at 1644 cm^−1^ assigned to the imine bond *(*C=N), confirmed that lipase was successfully anchored on the functionalized support (Figure 4 (Ae)).

From TG analysis (Figure 4 (Ba,b)), the thermal decomposition of hexadecyl groups from HDTMS gave a grafting density of 67 mg/g, higher than that of trimethyl groups from CTMS (45 mg/g) (Table 1). However, the corresponding surface coverage (S_cov_) obtained with HDTMS was extremely low (0.07 chain/nm^2^), indicating incomplete silylation of surface silanols (Table 1). Note that Sihgel@APTMS yielded a grafting density of 93 mg/g and a surface coverage of 0.28 chain/nm^2^, which are both lower than the values recorded on Sihgel@GPTMS (174 mg/g and 0.41 chain/nm^2^ respectively). Furthermore, the weight loss resulting from the thermal decomposition of the protein was found equal to 3.6%. Comparison of experimental and theoretical data (67 mg·g^−1^ vs. 85 mg·g^−1^) reveals that the hexadecyl groups provide only partial coverage of the hydrogel surface probably due to incomplete hydrolysis of the silane and insufficient interaction with silanols available on the support surface.

(c) Sihgel@CTMS@APTMS@CALB: Effect of the linker (glutaric anhydride vs. glutaraldehyde)

**Figure 5:**
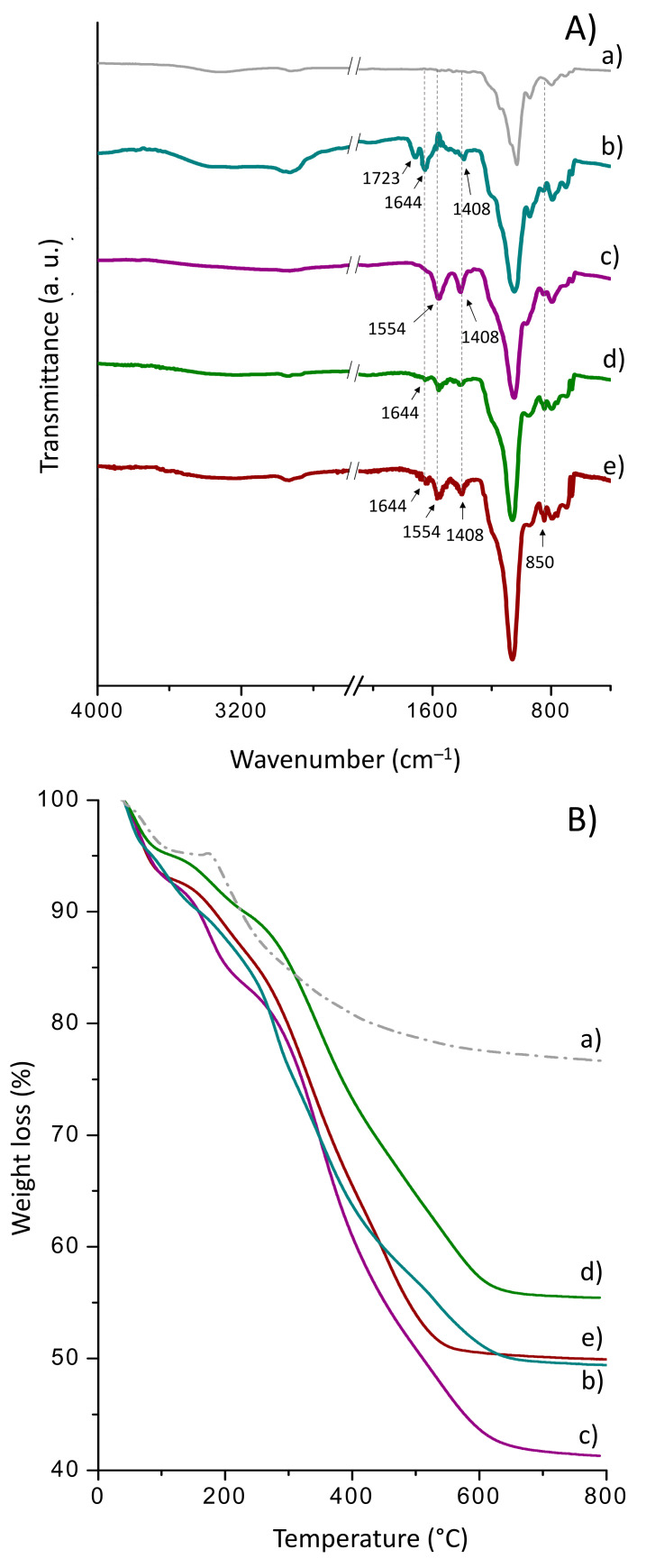
ATR-FTIR spectra (**A**) and TG curves (**B**) for (**a**) as-synthesized Sihgel, (**b**) Sihgel@CTMS@APTMS-GAC, (**c**) Sihgel@CTMS@APTMS-GAC@CALB, (**d**) Sihgel@CTMS@APTMS-GAH, and (**e**) Sihgel@CTMS@APTMS-GAH@CALB.

Activation of the primary amino groups with GAC resulted in the appearance of a new band at 1723 cm^−1^ assigned to the C=O stretch modes of aldehyde groups (Figure 5(Aa)). Moreover, reaction of both GAH and GAC with surface amino groups was confirmed by the CH_2_ bends at 1408 cm^−1^, as well as the C=N stretching modes at 1644 cm^−1^ (Figure 5(Aa,c)). Other bands at 2948 cm^−1^ and 2857 cm^−1^ on Sihgel@CTMS@APTMS@GAC can be ascribed to aldehyde C–H and alkyl C–H stretching vibrations, respectively. In all functionalized solids, the band at 850 cm^−1^ corroborates with the Si–C stretching vibration of –O–SiCH_3_ end groups from CTMS. Moreover, lipase immobilization on both supports was confirmed by the vibration at 1554 cm^−1^ assigned to the N–H bending of free amines (Figure 5(Ab,d)).

From the thermogravimetric curves (Figure 5B), the effective weight loss on Sihgel@CTMS@APTMS-GAC was determined to be 21.4%, which was almost 3.2%, higher than that recorded on Sihgel@CTMS@APTMS-GAH (18.1%). Such a difference is probably due to the higher crosslinking ability of GAC with respect to GAH, which may also explain its higher enzyme-loadings capacity (6.5% vs. 5.4%) (Figure 5(Bb,d) and Table 1).

## 3. Catalytic Activity

The oxidation of DFF to FDCA proceeds via a chemoenzymatic cascade reaction involving two consecutive steps. First, the lipase CALB catalyzes the perhydrolysis of ethyl acetate with hydrogen peroxide yielding peracetic acid. Then, the peracid oxidizes in situ the aldehyde group of DFF to carboxylic acid yielding the 5-formylfuran-2-carboxylic acid (FFCA), which is ultimately converted to FDCA (Table 2). In all our experiments, reaction was assessed by adding 2.0 equivalents H_2_O_2_ (30% *v*/*v*) in a mixture of ethyl acetate (EtOAc) and *tert*-butanol (tBuOH) (1:1 *v*/*v*) containing 10 mM DFF. Reaction products were analyzed using ^1^H NMR and HPLC. tBuOH was used as a solvent to avoid deactivation of CALB because of the high acidity of the acyl donor (EtOAc). Indeed, in the oxidation of furfural, Krystof et al. [51] have shown that reactions conducted in neat EtOAc, acting both as acyl donor and solvent, can lead to lower yields in furoic acid with respect to reaction performed in a tBuOH/EtOAc mixture.

Under those conditions, free CALB was not active (entry 1). However, when 2 mg lipase was immobilized by ionic bonding on Sihgel@CTMS (acetate buffer pH 4.6), 88% DFF conversion and 19% FDCA yield could be achieved (entry 2). Notably, DFF conversion was complete when the lipase loading was increased to 4 mg, giving 72% yield in FDCA (entry 3). When CALB was covalently attached to Sihgel@CTMS@GPTMS prepared with different GPTMS/SiO_2_ and CTMS/SiO_2_ molar ratios (entries 4 to 10), the immobilization rates were in the range of 89 to 97%, confirming the effectiveness of the epoxy ring as a reactive group for covalent anchoring of the protein. Notably, the DFF conversions achieved with 2 mg CALB were complete with all catalysts, while the FDCA yields were found to be dependent on GPTMS loading, decreasing from 42 to 34% when the GPTMS/SiO_2_ molar ratio was increased from 0.68 to 1.50 (entries 4–6). In particular, with 4 mg CALB, the FDCA yields could reach 70% and 51% using GPTMS/SiO_2_ molar ratios of 0.68 (entry 7) and 0.34 (entry 8) respectively. Similarly, increasing the CTMS/SiO_2_ ratio from 0.33 to 0.66 resulted in a drop of the FDCA yield from 70% (entry 7) to 41% (entry 9). However, a FDCA yield of 72% could be achieved by increasing the lipase loading to 8 mg, (entry 10). Taking into account the best performance/cost ratio, Sihgel@CTMS0.33@GPTMS0.68@CALB2 was found to be the most efficient bioreactor, giving a specific activity of 20.8 µmol g^−1^ min^−1^, a TTN of 288.8 mol^−1^ and a TOF of 41.3 min^−1^ (entry 4). Taken together, these results demonstrated that simultaneous functionalization of the silicified hydrogel with GPTMS and CTMS is an effective way for covalent anchoring and interfacial activation of the lipase.

The effect of hydrophobic groups on enzyme activity was established by comparing trimethyl- and hexadecyl- functionalized materials at the same silane/SiO_2_ molar ratio of 0.33. Regardless the immobilization approach (physisorption or covalent bond), the enzyme loadings on Sihgel@HDTMS ranged from 96% to 99% (entries 11 to 13, 15). Notably, the FDCA yield increased from 17% (entry 11) to 50% (entry 12) when CALB loading was increased from 2 mg to 4 mg. It is worth noting that although HDTMS acts as an effective hydrophobic agent, with 4 mg CALB, its effect on the interfacial activation of lipase seems to be less pronounced than that of CTMS (50% vs. 72% FDCA yields) (entries 12, 3). Accordingly, the bifunctional Sihgel@HDTMS@CTMS@CALB4 biocatalyst containing equimolar amounts of CTMS and HDTMS achieved 60% yield in FDCA (entry 13), which represents an intermediate value between CTMS (72%, entry 3) and HDTMS (50%, entry 12) taken separately.

The grafting density cannot explain such difference because it is slightly lower in the CTMS-functionalized material compared to the HDTMS-functionalized one (45 mg/g vs. 67 mg/g, Table 1). Conversely, the surface coverage with hexadecyl chains was found to be almost four times lower than that obtained with trimethyl groups (0.07 chains/nm^2^ vs. 0.28 chains /nm^2^). Therefore, the partial coverage of the hydrogel surface with hexadecyl moieties appears to be inadequate for the interfacial activation of lipase. Notably, grafting of CALB on the non-hydrophobisized support gave negligible DFF conversion (44%) and could not lead to the final product (0% FDCA yield) (entry 14), while 85% DFF conversion and 13% FDCA yield could be achieved with the Sihgel@HDTMS0.33@APTMS-GAH@CALB2 biocatalyst (entry 15). Taken together, these data indicate that the hydrophobic moieties of the silane and their surface coverage play a key role in optimal activation of the lipase.

On the other hand, comparison of Sihgel@CTMS@APTMS-GAC@CALB2 (entry 16) and Sihgel@CTMS@APTMS-GAH@CALB2 (entry 17) revealed that glutaric anhydride was a less efficient linker than glutaraldehyde (67% vs. 41 % FDCA yields), although higher grafting densities were obtained with the former (223 mg/g vs. 184 mg/g). This suggests that the CAG-activated chains resulting from succinylation of both amino groups and hydroxy amino acids from protein [52] may probably alter the proper functioning of lipase by masking the hydrophobic trimethyl groups, which are essential for interfacial activation of CALB. It is worth noting that the FDCA yield achieved with the Sihgel@CTMS@APTMS-GAH@CALB2 catalyst (entry 17) was also higher than that obtained with Sihgel@CTMS@GPTMS@CALB2 (entry 4) giving a specific activity of 33.2 µmol g^−1^ min^−1^, a TTN of 460.6 mol^−1^, and a TOF of 65.8 min^−1^.

These observations highlight the important role played by both grafting and hydrophobic groups on the catalytic performance of immobilized CALB. Indeed, the steric hindrance exerted by the thick layer resulting from crosslinking reactions of the epoxy ring [40,41] may cause conformation constraints on immobilized enzyme, restricting access of substrate to the active site. Conversely, partial covering of hydrogel surface with hardly hydrolysable hexadecyltrimethoxysilane seems not being adequate for the interfacial activation of lipase. Therefore, a proper balance needs to be found between chemically reactive groups and hydrophobic functions for an optimal functioning of the biocatalyst.

## 4. Recyclability

**Figure 6:**
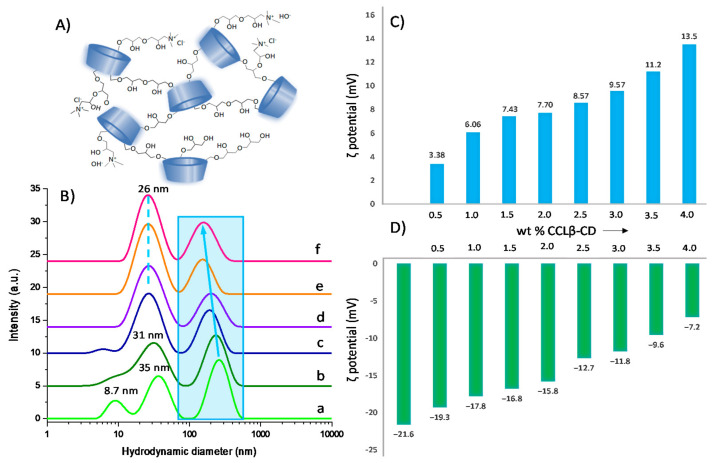
Schematic structure of cross-linked β-cyclodextrin oligomers (**A**); DLS data of mixtures prepared with 2 mg/mL CALB and different wt. % of CCLβ-CD (a: 0%; b: 0.5%; c: 1.5%; d: 2.5%; e: 3.0%; f: 4.0%) (**B**). Zeta potential of aqueous solutions prepared increasing wt. % of CCLβ-CD without (**C**) and with 2 mg/mL CALB (**D**).

The ability to recycle and reuse the catalyst is one of the most important criteria for industrial applications. Unfortunately, our most efficient catalyst, i.e., Sihgel@CTMS@APTMS-GAH@CALB2, could not be reused under the employed reaction conditions since the lipase completely lost its catalytic activity in the second run. Given that the furanic compounds formed during this reaction (FFCA and FDCA) bear carboxylic acid moieties in their cycle, they are likely to lower the pH in the vicinity of the enzyme, thereby creating an acidic microenvironment that can inhibit the lipase activity [53]. In fact, the pH of reaction medium was found to decrease at the end of the catalytic test from 4.8 to 3.1. To circumvent limitations due to pH gradients and assess the recycling potential of the immobilized CALB, we considered covering our biocatalyst with the cationic cross-linked β-cyclodextrin (CCLβ-CD) employed as a lipase-stabilizing agent. The polymeric CD network is obtained by crosslinking native β-CD with epichlorohydrin (EP) in the presence of a cationizing agent, the glycidyltrimethylammonium chloride (GTMAC) [54]. CCLβ-CD has received great interest as high performant carrier in drug [55,56] and gene delivery systems [57], as well as in heterogeneous catalysis [58]. Because this CD-based polymer contains quaternary ammoniums on its structure (Figure 6A), it should be able to protect the enzyme against pH variations, thus acting as a “solid buffer”.

Under the immobilization conditions employed, at pH below the isoelectric point of CALB (acetate buffer, pH 4.6), the presence of positive charges on CCLβ-CD can result in favorable electrostatic interactions with the negatively charged enzyme. Evidence for such interactions was provided by DLS measurements. Thus, at 25 °C, CALB solution (2 mg/mL) displayed three size populations that could be attributed to free enzyme (8.7 nm) and its aggregates (35 nm and 260 nm) (Figure 6B). Upon addition of CCLβ-CD up to 4 wt %, the size of the CALB aggregates decreased from 260 to 160 nm as a result of their dissociation after interaction with the cationic polymer. Notably, the size population at 8.7 nm corresponding to free enzyme totally disappeared at the expense of CALB/CCLβ-CD assemblies with an average diameter of 25–30 nm. Zeta potential measurements also confirmed interaction between the two entities. For instance, in the enzyme-free CCLβ-CD solution (Figure 6C), ζ potential increased from +3.38 to +13.5 mV with polymer amount, while negative values were obtained in the CALB/CCLβ-CD mixture, increasing from −21.6 to −7.2 mV with CCLβ-CD loading. Overall, our results confirm that electrostatic interactions take place between CALB and CCLβ-CD in aqueous solution, yielding stable supramolecular assemblies.

**Figure 7:**
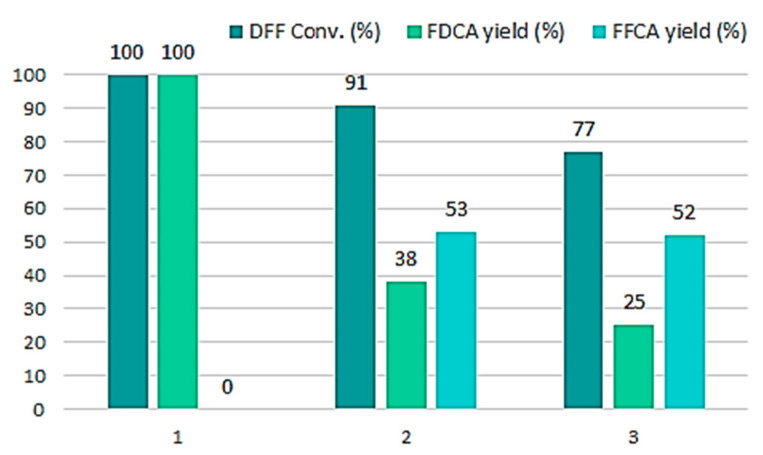
Catalytic recyclability of SiO_2_@CTMS@APTMS-GAH@CALB@CCLβ-CD biocatalyst. Reaction conditions: 10 mM DFF, 2 mL EtOAc/tBuOH (1:1 *v*/*v*), sequential addition of 2.0 eq. H_2_O_2_ (30% *v*/*v*) every hour for seven hours, temperature 40 °C, reaction time 24 h.

With a surface coverage of 2 wt% in CCLβ-CD, the recyclability of the most efficient nanoreactor, i.e., Sihgel@CTMS@APTMS-GAH@CALB was then evaluated and the activities obtained across three consecutive runs are shown in Figure 7. Although a drop in the FDCA yield was noticed from 100% to 38% in the second run and to 25% in the third one, the DFF conversions were still high (77%), with FFCA being the only intermediate (52%). We explain such a decrease in the FDCA yield by the large number of small particles that were lost at the very first rinsing step, causing a decrease of the amount of lipase remaining in the reaction mixture. In fact, the silicified hydrogel is very polydisperse in size. It is composed of very small particles originating from free (uncomplexed) cyclodextrin and larger ones formed by silicification of poly*pseudo*rotaxanes and poly*pseudo*rotaxane-based nanocrystallites. All of these species are present in the starting F127/α-CD hydrogel and are replicated after silicification producing a wide range of particle sizes, which are at the origin of the microstructure heterogeneity. At the end of the first run, we observed an important decrease in the amount of residual solid, which was actually almost half lower (10.5 mg) than that introduced in the reaction medium (20.8 mg), then a stabilization to 8.4 mg in the second run and to 7.9 mg in the third one. This suggests that almost all tiny particles were removed during the first rinsing step. However, we also noticed that the rinsing solution was able to catalyze effectively the DFF oxidation, achieving conversions close to 100% (Figure S2, ESI). Given that free CALB was not active in this reaction (entry 1, Table 2), this in an indication that enzyme leaching that may result from the hydrolysis of the imine bond should not be significant under these modified conditions. Overall, these results confirm that CCLβ-CD is a good stabilizing agent for our biocatalyst since it effectively protects the enzyme against deactivation under acidic conditions. Nevertheless, CCLβ-CD/CALB molar ratio in the nanoreactor is an important parameter that needs to be further investigated in the future in order to optimize the effectiveness of recycling process.

## 5. Conclusions

In summary, we have shown that the hierarchically porous silica monoliths obtained by silicification of a supramolecular Pluronic F127/α-cyclodextrin hydrogel act as a promising host matrices for the immobilization of lipase B from *Candida antarctica*. Functionalization of the silicified hydrogel surface with chemically reactive groups (epoxide and primary amine) and hydrophobic functions (trimethyl and hexapropyl) permitted covalent anchoring and interfacial activation of the lipase. Comparison of the catalytic activities of different catalysts in the oxidation of 2,5-diformylfuran demonstrated the necessity to find a balance between the density of surface coverage and hydrophobic chain length. Therefore, the dense organic layer obtained with (3-glycidiloxypropyl) trimethoxysilane resulting from crosslinking reactions of the epoxy ring was found to be detrimental for lipase activity. Similarly, using glutaric anhydride (GAC) as a spacer on the (3-aminopropyl) trimethoxysilane-functionalized material caused a decline in enzyme activity with respect to glutaraldehyde. On the other hand, the hydrophobic chlorotrimethylsilane with a shorter chain length was found to provide better coverage of the hydrogel surface compared to hexadecyltrimethoxysilane and was more efficient for the interfacial activation of CALB. Among the different biocatalysts, Sihgel@CTMS@APTMS-GAH@CALB gave the best catalytic performance in the oxidation of DFF, achieving full DFF conversion with 67% FDCA yield after 7 h at 40 °C and almost quantitative FDCA yield after 24 h. While immobilized CALB was completely deactivated after the first run, the use of cationic cross-linked β-cyclodextrin as a stabilizing agent permitted to significantly increase the operational stability of lipase, enabling efficient recycling and reuse of the biocatalyst in at least three cycles. This study opens up new perspectives on the use of hierarchically porous supramolecular hydrogels in construction of other chemoenzymatic cascades integrating enzymatic and heterogeneous catalysts for spatially confined chemo-enzymatic reactions.

## 6. Experimental

### 6.1. Materials

Lipase B from Candida antarctica (CALB), recombinant from Aspergillus oryzae (Mw *33* kDa) was purchased from Sigma Aldrich (Saint-Quentin-Fallavier, France). CALB contains 317 amino acid residues [59] and its active site is composed of a catalytic triad consisting of nucleophilic serine, histidine, and aspartate or glutamate [60]. Pluronic F127 [PEO_100_PPO_70_PEO_100_ where PEO stands for poly(ethylene oxide) and PPO for poly(propylene oxide), average Mw 12,500 g/mol], tetramethyl orthosilicate (TMOS 98%, Mw 152.22 g/mol), chlorotrimethylsilane (CTMS > 98%, Mw 108.64 g/mol), hexadecyltrimethoxysilane (HDTMS > 85%, Mw 346.6 g/mol), (3-aminopropyl)-trimethoxysilane (APTMS 97%, Mw 221.37 g/mol), (3-Glycidyloxypropyl)trimethoxysilane (GPTMS, ≥ 98%, Mw 236.34 g/mol), glutaraldehyde (GAH, 50% in H_2_O, Mw 100.11 g/mol), glutaric anhydride (GAC, 95%, Mw 114.1 g/mol), N-(3-Dimethylaminopropyl)-N′-ethylcarbodiimide hydrochloride (EDC hydrochloride > 98%, Mw 191.7 g/mol), 2,5-diformylfuran (DFF 97%, Mw 124.1 g/mol), 2,5-furandicarboxylic acid (FDCA 97%, Mw 156.1 g/mol), 5-formylfuran-2-carboxylic acid (FFCA, Mw 140.1 g/mol), phosphate buffer solution (50 mM, pH 7.5), ethylacetate (EtOAc), and tert-butanol (t-BuOH) were purchased from Sigma Aldrich (Saint-Quentin-Fallavier, France). Acetate buffer solution (pH 4.6) was procured from Honeywell Fluka (Illkirch, France). Native α-cyclodextrin (α-CD, 99%, Mw 972.85 g/mol) was procured from Wacker Chemie GmbH (Lyon, France) while *ca*tionic cross-linked β-CD (CCLβ-CD, Mw ~20 kg/mol) was a gift from Roquette Frères (Lestrem, France). The ^1^H NMR and ^13^C NMR spectra of polymer in D_2_O are shown in Figure S3, ESI. All chemicals were used as received, without further purification.

### 6.2. Preparation of Silicified Hydrogel

The silicified hydrogel (denoted Sihgel) was prepared according to a previously reported method with some modifications [30]. Briefly, 2.0 g of α-CD (100 mg/mL) was introduced into 20 mL of a micellar F127 solution (25 mg/mL of Pluronic F127 in water). The mixture was stirred at room temperature for 15 min, then stored in a closed vial at 4 °C for 24 h until a water-swollen gel formed. The pH of the hydrogel was measured to be 6.3. Subsequently, 1.42 g of TMOS (α-CD/TMOS molar ratio = 0.22) was added dropwise to the hydrogel and maintained under stirring (800 rpm) at room temperature for 2 h. The mixture was then loaded into a 40-mL Teflon-lined autoclave and heated at 60 °C for 48 h to complete the condensation reaction between silanols. The excess of hydrogel was removed by washing several times with water and ethanol.

### 6.3. Functionalization of Silicified Hydrogel

Prior to functionalization with different reactive and hydrophobic groups, 250 mg of the silicified hydrogel (Sihgel) was dried under vacuum conditions at 180 °C overnight in order to remove any traces of solvent adsorbed into the pores.

(i) Sihgel@CTMS@GPTMS. Surface functionalization with GPTMS and CTMS was carried out according to a method reported earlier by Renard et al. [61] with some modifications. Typically, 250 mg of dried Sihgel was introduced in 25 mL toluene together with 175 μL CTMS (0.054 M, CTMS/SiO_2_ = 0.33) and 750 μL GPTMS (0.113 M, GPTMS/SiO_2_ = 0.68). The mixture was maintained under reflux at 100 °C for 16 h. Reaction was performed in toluene because non-polar solvents have been reported to facilitate aggregation of silane ligands on the silica surface, thus favoring interaction with silanol groups [48,62]. The solid was collected by centrifugation, then washed with water and ethanol and finally dried under vacuum conditions at 25 °C for 10 h. GPTMS/SiO_2_ molar ratio was varied between 0.34 and 1.5 while CTMS/SiO_2_ one was fixed to 0.33 or 0.66.
(ii) Sihgel@HDTMS@APTMS-GAH. Surface functionalization with APTMS was carried out according to two methods reported earlier on conventional silica by Sorensen et al. [63] and Kao et al. [64] with some modifications. Typically, 250 mg of silicified hydrogel was suspended in 25 mL of anhydrous toluene, followed by successive addition of 630 µL HDTMS (0.055 M, HDTMS/SiO_2_ = 0.33) and 122 µL APTMS (0.027 M, APTMS/SiO_2_ = 0.16). Because of its high degree of hydrophobicity, HDTMS was hydrolyzed first in an oxalic acid solution (0.1 M oxalic acid) before grafting. After refluxing at 100 °C for 24 h, the solid denoted Sihgel@HDTMS@APTMS was collected by centrifugation, washed several times with toluene, then dried under vacuum and finally stored under inert (N_2_) atmosphere. Subsequently, 50 mg of the functionalized Sihgel*@*HDTMS@APTMS material was dispersed in 1.5 mL acetate buffer (pH 4.6) and 77 µL glutaraldehyde (GAH) was added (GAH/APTMS = 3.1). After stirring at room temperature for 10 h, the GAH cross-linked material (denoted Sihgel@HDTMS@APTMS-GAH) was collected by centrifugation, then washed with water and ethanol and finally dried under vacuum conditions at 25 °C for 10 h.
(iii) Sihgel@CTMS@APTMS-GAH(GAC). Typically, 250 mg silicified hydrogel was suspended in 25 mL of anhydrous toluene, followed by successive addition of 175 µL CTMS (0.054 M, CTMS/SiO_2_ = 0.33) and 122 µL APTMS (0.027 M, APTMS/SiO_2_ = 0.16). After refluxing at 100 °C for 24 h, the solid denoted Sihgel@CTMS@APTMS was collected by centrifugation, washed several times with toluene, then dried under vacuum and stored under inert (N_2_) atmosphere. Subsequently, 50 mg of the functionalized Sihgel@CTMS@APTMS material was dispersed in 1.5 mL acetate buffer (pH 4.6) and 77 µL of glutaraldehyde (GAH) was added (GAH/APTMS = 3.1). After stirring at room temperature for 10 h, the GAH cross-linked material (denoted Sihgel*@*CTMS@APTMS-GAH) was collected by centrifugation, then washed with water and ethanol and finally dried under vacuum conditions at 25 °C for 10 h. In another synthesis, glutaric anhydride (GAC) was employed as a linker. Typically, 50 mg of Sihgel@CTMS was dispersed in 1.5 mL DMF, then 50 mg·GAC was added (GAC/APTMS = 3.2). The mixture was stirred at room temperature for 1 h, then refluxed at 60 °C for 9 h. The solid was recovered by centrifugation, then washed with water and ethanol and finally dried under vacuum.

In some experiments, for comparison purposes, the Sihgel matrix was silanized with hydrophobic groups from CTMS (Sihgel@CTMS) and HDTMS (Sihgel@HDTMS) taken separately, before considering their combine use (Sihgel@CTMS@HDTMS). A non-hydrophobized surface was also prepared for a control experiment by functionalizing Sihgel with APTMS followed by the activation with GAH (Sihgel@APTMS-GAH). The CTMS/SiO_2_ and HDTMS/SiO_2_ molar ratio used was 0.33 unless stated otherwise.

### 6.4. Immobilization of Lipase B from Candida Antarctica

For the immobilization of CALB, 2 mg of lipase solubilized in 0.3 mL buffer solution was added to 20 mg of Sihgel@CTMS@GPTMS dispersed by ultrasound in 1.2 mL phosphate buffer. Immobilization was carried out at pH 7.5 because the epoxide-ring opening is greatest under near neutral or moderate alkaline conditions [41]. After stirring in an ice bath for 16 h, the supported biocatalyst was collected by centrifugation, washed several times with water and ethanol, then dried under vacuum. The same immobilization procedure was applied on Sihgel@HDTMS@APTMS-GAH and Sihgel@CTMS@APTMS-GAH(GAC) with the only exception that phosphate buffer (pH 7.5) was replaced by acetate buffer (pH 4.6). In fact, the formation of the imine bond is generally favored near a pH of 5.0 [42]. In the case where GAC was used as linker, 10 mg of 1-(3-diméthylaminopropyl)-3-éthylcarbodiimide (EDC) were added for carboxylic acid activation in GAC. For comparison purposes, CALB was also anchored by ionic bonding on hydrophobized Sihgel. Typically, 2 mg of CALB was first dissolved in 1.5 mL acetate buffer solution (pH 4.6), then 20 mg of Sihgel@CTMS (or Sihgel@HDTMS) was added. The pH of reaction medium was chosen between the isoelectric point of CALB (pI 6) and the point of zero charge of silica (PZC 2.0–3.5), so that attractive electrostatic interactions can occur between the support and the enzyme. After stirring in an ice bath for 16 h, the supported biocatalyst was collected by centrifugation, washed with water and ethanol, then dried under vacuum.

### 6.5. Characterization Methods

Attenuated total reflexion Fourier transform infrared (ATR-FTIR) measurements were performed using a MIRacle Diamond prism on a Shimadzu IR Prestidge-21 spectrometer. All spectra were recorded with a resolution of 2 cm^−1^ in the 4000–400 cm^−1^ region. Thermogravimetric (TG) analyses were carried out in platinum crucibles using a Setaram TG-DTA 92 microbalance. Samples were analyzed in duplicate. All measurements were performed in air at a flow rate of 20 mL/min using a heating ramp of 8 °C/min, from 40 to 800 °C. The surface coverage of the silanized materials (S_cov_) (chain/nm^2^) was determined based on the following equation as described by Chevigny et al. [65]

(1) $$S_{\mathrm{cov}}=\left(\frac{S_{\mathrm{BET}}}{M_{\mathrm{gr}}{\;\times \;N}_{A}}\right)\;\times \;\left(\frac{W_{\mathrm{tot}}-{\;W}_{\mathrm{ref}}\;}{100-\left(W_{\mathrm{tot}}-{\;W}_{\mathrm{ref}}\right)}\right)$$

where S_BET_ is the specific surface area of the support (nm^2^/g), N_A_ is the Avogadro’s constant (6.02 × 10^23^ /mol), M_gr_ is the molar weight of the functional group, W_tot_ and W_ref_ are the weight losses of functionalized silica and bare silica respectively. Nitrogen adsorption-desorption isotherms were collected at −196 °C using a Micromeritics Tristar 3020. Typically, prior to analysis, 40–60 mg samples were outgassed at 180 °C for 16 h to remove species adsorbed on the surface. The specific surface area (S_BET_) was calculated from N_2_-adsorption isotherms using the Brunauer-Emmet-Teller (BET) method. Pore size distributions and pore volumes were determined using the BJH method assuming a cylindrical pore structure. The maximum grafting density (D_g_^max^) (mg/g) was determined using the following equation, assuming that a monolayer of silane was formed on the surface of silanized hydrogel [48].

(2) $$D_{g}^{\max}=\left(\frac{S_{\mathrm{BET}}}{S_{\mathrm{Si}}}\right)\left(\frac{M_{W}}{N_{A}}\right)$$

where S_BET_ is the specific surface area of the silicified hydrogel (nm^2^/g), S_Si_ is the surface area occupied by a silane ligand (nm^2^), M_W_ is the organosilane molecular weight, and N_A_ is the Avogadro’s constant. Transmission electron microscopy (TEM) images were recorded on a TECNAI microscope operating at 200 kV. Powders were deposited directly on a carbon coated copper grid. Scanning electron microscopy (SEM) images were recorded at 3 keV with a FEG Hitachi S-4700 microscope. UV-Visible measurements were carried out using a Perkin Elmer (Lambda 19) spectrophotometer. Enzyme loading in the silicified hydrogel was determined by measuring the initial and final concentration of protein within the immobilization solution using the Bradford method [66]. Calibration curves were established by measuring the ratio between the absorbance at 595 nm corresponding to the anionic blue form of the Coomassie Brilliant Blue G-250 binding to the protein, and the absorbance at 450 nm corresponding to the cationic red form of the dye. The immobilization efficiency (%) and the loading capacity (wt. %) were deduced by mass balance using the following equations [67]:

(3) $$\;\mathrm{Immobilization}\;\mathrm{efficiency}=\frac{\left(m-C_{1}V_{1}\right)}{m}\times 100$$

(4) $$\mathrm{Loading}\;\mathrm{capacity}=\frac{\left(m-C_{1}V_{1}\right)}{\mathrm{Ws}}\times 100$$

where m (mg) is the mass of the enzyme added to the immobilization solution, C_1_ (mg/mL) and V_1_ (mL) are the concentration of enzyme in the supernatant and its volume respectively, and Ws (g) is the weight of the support. Polarized optical microscopy images were recorded on an Olympus (BX51) microscope. Viscosity measurements were carried out at 25 °C using a viscosimeter from Brookfield equipped with a cylindrical geometry (module SC4–18). The apparent viscosity vs. shear-rate plots were recorded in a 0.1–500 s^−1^ range of shear-rate. Dynamic light scattering (DLS) and Zeta potential measurements were performed at 25 °C using the Malvern Zeta Nanosizer Instrument equipped with a 4.0 mW He-Ne red laser operating at 633 nm. Detection was carried out in backscattering mode (scattering angle 173°) with respect to the incident beam. Each sample was analyzed three times with an average of ten runs per measurement.

### 6.6. Activity Measurements

Free CALB used in this study had a hydrolytic activity of 9 U/mg. One lipase unit corresponds to the amount of enzyme releasing 1 μmol of butyric acid per minute at 40 °C (pH 8.0) using tributyrin as substrate. The catalytic performance of immobilized lipase was evaluated in the oxidation of DFF to FDCA as reported by Qin et al. [68], with some modifications. Typically, DFF (2.48 mg, 10 mM) was dissolved in 2 mL of a mixture of ethyl acetate (EtOAc) and tert-butanol (t-BuOH) (1:1 *v/v*), to which 2.0 equivalents of aqueous H_2_O_2_ (4.5 µL, 30% *v/v*) were added. The reaction was started with addition of 2 mg of CALB and maintained under stirring in a thermostatic bath at 40 °C. About 2.0 equivalents H_2_O_2_ aliquots were added regularly every hour, up to 7 h of reaction time. The analysis of DFF and its oxidation products (FDCA and FFCA) was carried out using ^1^H nuclear magnetic resonance (^1^H NMR) spectroscopy and high liquid performance chromatography (HPLC). ^1^H NMR spectra were recorded on a BRUKER DPX300 Avance spectrometer operating at 300 MHz at 25 °C with 16 scans per measurement. Prior to analysis, products were dissolved in 600 µL DMSO. Typical profiles of the ^1^H NMR spectra of isolated products and reaction mixture are shown in Figure S4. DFF conversion (%) together with the FFCA and FDCA yields (%) was calculated according to the following equations:

(5) $$\mathrm{DFF}\;\mathrm{Conversion}=100-\left(\frac{A_{\mathrm{DFF}}/2}{A_{\mathrm{FDCA}}/2+A_{\mathrm{FFCA}}+A_{\mathrm{DFF}}/2}\times 100\right)$$

(6) $$\mathrm{FFCA}\;\mathrm{yield}=\frac{A_{\mathrm{FFCA}}}{A_{\mathrm{FDCA}}/2+A_{\mathrm{FFCA}}+A_{\mathrm{DFF}}/2}\times 100$$

(7) $$\mathrm{FDCA}\;\mathrm{yield}=\frac{A_{\mathrm{FDCA}}/2}{A_{\mathrm{FDCA}}/2+A_{\mathrm{FFCA}}+A_{\mathrm{DFF}}/2}\times 100$$

where A_FDCA_, A_FFCA_, and A_DFF_ are the integrated areas of peak a: δ = 7.30 ppm (s, 2H, Ar) from FDCA, peak b: δ = 7.60 ppm (d, 1H, Ar) from FFCA and peak a: δ = 7.67 ppm (s, 2H, Ar) from DFF, respectively. The factor 2 derives from the two protons on the furan ring of FDCA and DFF, whereas only one proton is available on the furan ring of FFCA. HPLC analyses were performed on a Perkin Elmer Flexar apparatus using an Aminex HPX-87H (300 mm length × 7.8 mm diameter) column heated at 60 °C. The mobile phase was acetic acid (0.2%) at a flow rate of 0.6 mL/min. Aliquots of 1 µL of each sample were injected and analyzed at a wavelength of 284 nm using a photodiode array detector. Standard calibration curves were used to determine the amounts of DFF, FFCA, and FDCA. HPLC calibration curves and typical chromatograms of the isolated products and reaction mixture are shown in Figures S5 and S6.

## Figures and Tables

**Table 1:** Parameters deduced from N_2_-adsorption and TG analysis for the silica materials before and after modification with different functional groups and lipase immobilization.

Sample	Total Weight Loss ^a^ (%)	Effective Weight Loss ^b^ (%)	D_g_^c^ (mg·g^−1^)	D_g_^max, d^ (mg·g^−1^)	S_cov_^e^ (chain·nm^−2^)
Sihgel@CTMS0.33	22.3	4.5	45	57	0.24
Sihgel@GPTMS0.68	35.2	17.4	174	105	0.41
Sihgel@CTMS0.33@GPTMS0.68	38.5	20.7	207	-	-
Sihgel@CTMS0.33@GPTMS0.68@CALB	41.0	2.5	25	-	-
Sihgel@HDTMS0.33	24.5	6.7	67	85	0.07
Sihgel@APTMS0.16	27.1	9.3	93	68	0.28
Sihgel@HDTMS@APTMS-GAH	31.1	13.3	133	-	-
Sihgel@HDTMS@APTMS-GAH@CALB	32.5	1.4	14	-	-
Sihgel@CTMS@APTMS-GAC	39.2	21.4	214	-	-
Sihgel@CTMS@APTMS-GAC@CALB	45.7	6.5	65	-	-
Sihgel@CTMS@APTMS-GAH	35.9	18.1	181	-	-
Sihgel@CTMS@APTMS-GAH@CALB	41.3	5.4	54	-	-

^a^ Weight loss between 180 and 700 °C, ^b^ weight loss corrected by the decomposition profile of sol-gel silica before surface functionalization, ^c^ grafting density of functional groups between 180 and 700 °C, ^d^ maximum grafting density determined from Equation (2) (see Experimental part), ^e^ surface coverage determined from Equation (1) (see Experimental part).

**Table 2:** Catalytic performance of lipase CALB immobilized in Sihgel modified with different functional groups in the oxidation of DFF to FDCA ^a^.

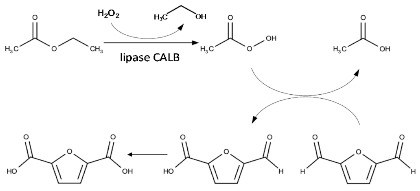									
Entry	Catalyst	Immobilization Efficiency (%) ^b^	Loading Capacity (wt.%) ^c^	DFF Conversion (%)	Yield (%)		Specific Activity (µmol g^−1^ min^−1^) ^g^	TTN (mol mol^−1^) ^h^	TOF (min^−1^) ^i^
					**FFCA (%)**	**FDCA (%)**			
**1**	free CALB	-	-	0	0	0	0	0	0
**2**	Sihgel@CTMS0.33 ^d^ @CALB2 ^e^	95	9.5	88	69	19	8.5	117.4	16.8
**3**	Sihgel@CTMS0.33@CALB4	95	19	100	28	72	18.0	250.1	35.7
**4**	Sihgel@CTMS0.33@GPTMS0.68@CALB2	96	9.6	100	58	42	20.8	288.8	41.3
**5**	Sihgel@CTMS0.33@GPTMS1.00@CALB2	97	9.7	100	61	39	19.1	265.4	37.9
**6**	Sihgel@CTMS0.33@GPTMS1.50@CALB2	97	9.7	100	66	34	16.7	231.3	33.0
**7**	Sihgel@CTMS0.33@GPTMS0.68@CALB4	89	17.8	100	30	70	18.7	259.6	37.1
**8**	Sihgel@CTMS0.33@GPTMS0.34@CALB4	96	19.2	100	49	51	12.6	175.3	25.0
**9**	Sihgel@CTMS0.66@GPTMS0.68@CALB4	92	18.4	100	59	41	10.6	147.1	21.0
**10**	Sihgel@CTMS0.66@GPTMS0.68@CALB8	96	38.4	100	28	72	8.9	123.8	17.7
**11**	Sihgel@HDTMS0.33@CALB2	96	9.6	82	65	17	6.9	95.8	13.7
**12**	Sihgel@HDTMS0.33@CALB4	99	19.8	100	50	50	12.0	166.7	23.8
**13**	Sihgel@HDTMS0.33@CTMS0.33@CALB4	98	19.6	100	40	60	14.6	202	28.9
**14**	Sihgel@APTMS0.16-GAH@CALB4	89	8.9	44	44	0	0.0	0.0	0.0
**15**	Sihgel@HDTMS0.33@APTMS0.16-GAH@CALB2	96	9.6	85	72	13	5.5	76.0	10.9
**16**	Sihgel@CTMS0.33@APTMS0.16-GAC@CALB2	95	9.5	100	59	41	20.6	284.8	40.7
**17**	Sihgel@CTMS0.33@APTMS0.16-GAH@CALB2	96	9.6	100	33	67	33.2	460.6	65.8
**18**	Sisg ^f^ @CTMS0.33@APTMS0.16-GAH@CALB2	72	7.2	24	24	0	0.0	0.0	0.0

^a^ Reaction conditions: 10 mM DFF, 2 mL EtOAc/tBuOH (1:1. *v*/*v*), sequential addition of 2.0 equivalents aqueous H_2_O_2_ (30% *v*/*v*) every hour for seven hours, temperature 40 °C, reaction time 7 h. ^b^ Calculated from Equation (3) (see Experimental part). ^c^ Calculated from Equation (4) (see Experimental part). ^d^ CTMS/SiO_2_ molar ratio = 0.33. ^e^ 2 mg CALB in 20 mg support (1.33 mg/mL CALB in the immobilization solution). ^f^ template-free sol-gel silica. ^g^ specific activity was calculated as µmol of product formed (two µmol peracid per µmol of FDCA) per g of protein in one minute. ^h^ TTN = moles of FDCA formed divided by moles of protein. ^i^ TOF = TTN divided by reaction time.

## Data Availability

Not applicable.

## Supplementary Materials

The following are available online at https://www.mdpi.com/article/10.3390/gels8010003/s1. Figure S1: (A) Viscosity vs. shear-rate curves for different mixtures prepared with 100 mg/mL α-CD and increasing concentrations of pluronic F127: (a) 8 mg/mL, (b) 16 mg/mL and (c) 30 mg/mL. All measurements were performed at 25 °C. (B) Visual aspect of the supramolecular F127/α-CD hydrogel (16 mg/mL F127) before and after shaking. Figure S2: ^1^H NMR (300MHz, DMSO-D6) spectrum of the reaction products obtained on DFF oxidation catalyzed by the supernatant recovered after the first run with the Sihgel@CTMS@APTMS-GAH@CALB2 biocatalyst. Reaction conditions: 10 mM DFF, 2 mL EtOAc/tBuOH (1:1. *v/v*), sequential addition of 2.0 equivalents aqueous H_2_O_2_ (30% *v/v*) every hour for 7 h, temperature 40 °C, reaction time 24 h. Figure S3: ^1^H NMR (300MHz, D_2_O) (A) and ^13^C NMR (D_2_O, 12,000 accumulations) (B) spectra of cationic cross-linked β-cyclodextrin (CCLβ-CD). The signal at 3.16 ppm in (A) is characteristic of the methyl protons from trimethylammonium groups. The resonance a in (B) is typical of C7, C8, C7′, C8′, and C9′ from the 2-hydroxypropyl ether segments. Resonances C8” and C9” indicate also the presence of glycidyl group which may be responsible for gelation of CCLβ-CD after prolonged storage. Figure S4: ^1^H NMR (300MHz, DMSO-D6) spectra of (a) DFF a: δ = 7.67 (s, 2H, Ar), b: δ = 9.82 (s, 2H, aldehyde); (b) FFCA a: δ = 7.39 (d, 1H, Ar), b: δ = 7.60 (d, 1H, Ar), c: δ = 9.73 (s, 1H, aldehyde); (c) FDCA a: δ = 7.30 (s, 2H, Ar); (d) Typical spectrum of reaction products obtained with Sihgel@CTMS@APTMS-GAH@CALB2: DFF conversion 100%; FFCA yield 10% and FDCA yield 90%. Figure S5: HPLC chromatograms of standard solutions: (a) DFF; (b) FFCA; (c) FDCA and (d) typical spectrum of the reaction mixture. Analytical conditions: mobile phase acetic acid (0.2%), temperature 60 °C, flow rate 0.6 mL/min. UV detection at 284 nm. Figure S6: HPLC calibration curves of (a) DFF, (b) FFCA and (c) FDCA. The analytical conditions were the same as those described in Figure S5.

## Abbreviations

CD	Cyclodextrin
Pluronic F127	PEO_100_PPO_70_PEO_100_ PEO: poly(ethylene oxide) and PPO: poly(propylene oxide)
CALB	Lipase B from *Candida antarctica*
TMOS	Tetramethyl orthosilicate
CTMS	Chlorotrimethylsilane
HDTMS	Hexadecyltrimethoxysilane
APTMS	(3-aminopropyl)-trimethoxysilane
GPTMS	(3-Glycidyloxypropyl)trimethoxysilane
GAH	Glutaraldehyde
GAC	Glutaric anhydride
EDC	N-(3-Dimethylaminopropyl)-N′-ethylcarbodiimide hydrochloride
CCLβ-CD	Cationic cross-linked β-CD
EP	Epichlorohydrin
GTMAC	Glycidyltrimethylammonium chloride
DFF	2,5-diformylfuran
FDCA	2,5-furandicarboxylic acid
FFCA	5-formylfuran-2-carboxylic acid
DLS	Dynamic Light Scattering
S_BET_	Surface area determined by the Brunauer, Emmett and Teller method
BJH	Barrett, Joyner and Halenda
TEM	Transmission Electron Microscopy
SEM	Scanning Electron Microscopy
NMR	Nuclear Magnetic Resonance
HPLC	High Performance Liquid Chromatography
ATR-FTIR	Attenuated Total Reflexion Fourier Transform Infrared spectroscopy
TGA	Thermogravimetric analysis
D_g_	Grafting density
D_g_^max^	Maximum grafting density
S_cov_	Surface coverage
