# Supplementary material for: Effect of Functional Group on the Catalytic Activity of Lipase B from Candida antarctica Immobilized in a Silica-Reinforced Pluronic F127/α-Cyclodextrin Hydrogel
Source: Gels. 2021 Dec 21;8(1):3. doi: 10.3390/gels8010003 (PMC8775079; doi:10.3390/gels8010003)
Supplement: Supplementary file 1 [file gels-08-00003-s001.zip › gels-1481197-supplementary-done.pdf]

gels

Electronic Supplementary Information for

Effect of Functional Group on the Catalytic Activity of Lipase B from *Candida antarctica* Immobilized in a Silica-Reinforced Pluronic F127/α-Cyclodextrin Hydrogel

Cédric Decarpigny [1], Anne Ponchel [1], Eric Monflier [1] and Rudina Bleta [1,*]

[1] Univ. Artois, CNRS, Centrale Lille, ENSCL, Univ. Lille, UMR 8181-UCCS-Unité de Catalyse et Chimie du Solide, F-62300 Lens, France; cedric.decarpigny@univ-artois.fr (C.D.); anne.ponchel@univ-artois.fr (A.P.); eric.monflier@univ-artois.fr (E.M.); rudina.bleta@univ-artois.fr (R.B.)

* Correspondence: rudina.bleta@univ-artois.fr; Tel.: (+33) 3 21 79 17 40

(A)

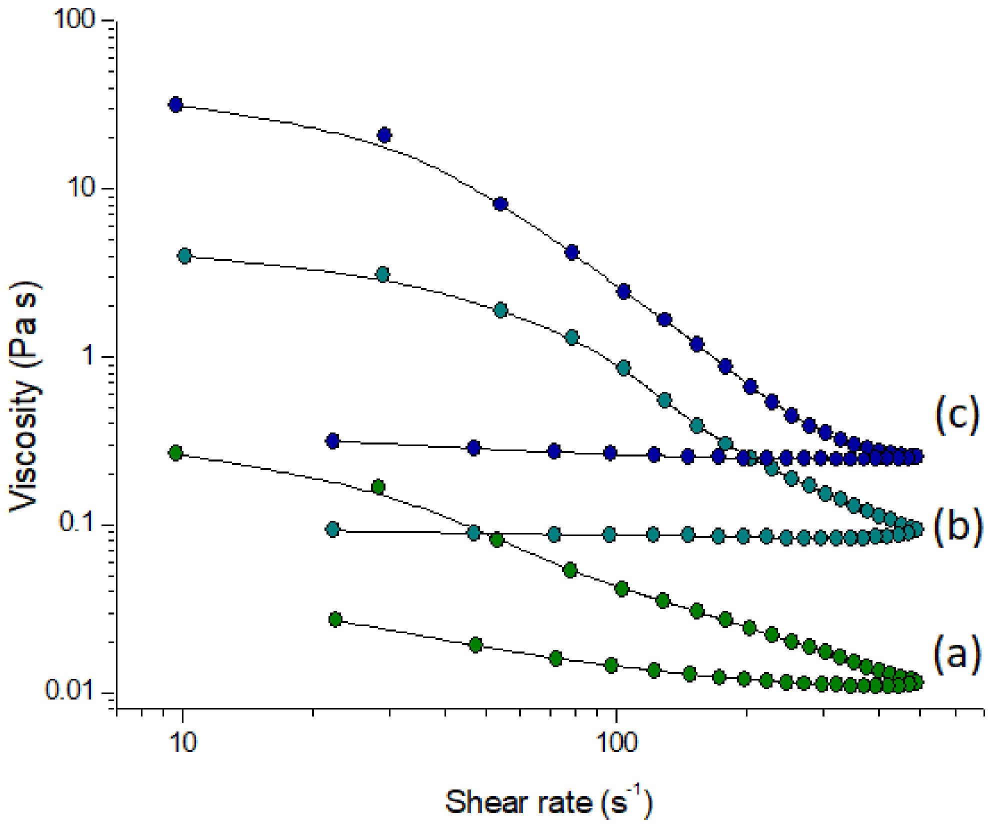

(B)

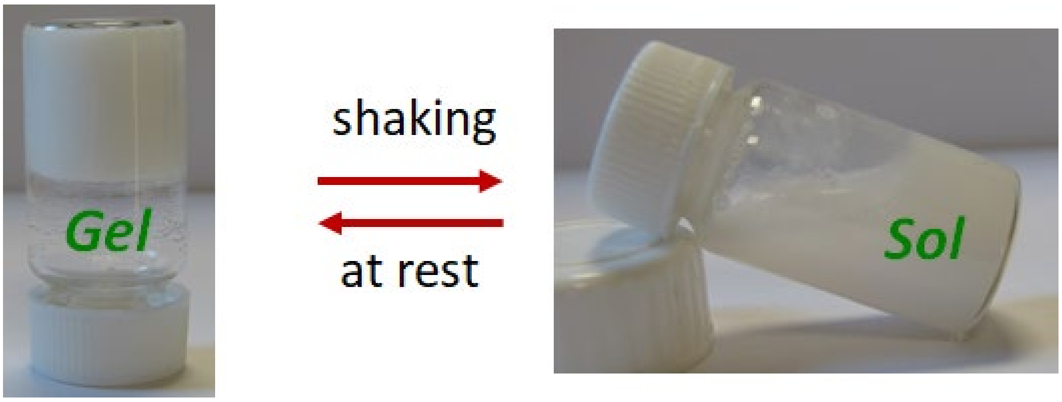

Figure S1. *(A) Viscosity vs. shear-rate curves for different mixtures prepared with 100 mg/mL α-CD and increasing concentrations of pluronic F127: (a) 8 mg/mL, (b) 16 mg/mL and (c) 30 mg/mL. All measurements were performed at 25 °C. (B) Visual aspect of the supramolecular F127/α-CD hydrogel (16 mg/mL F127) before and after shaking.*

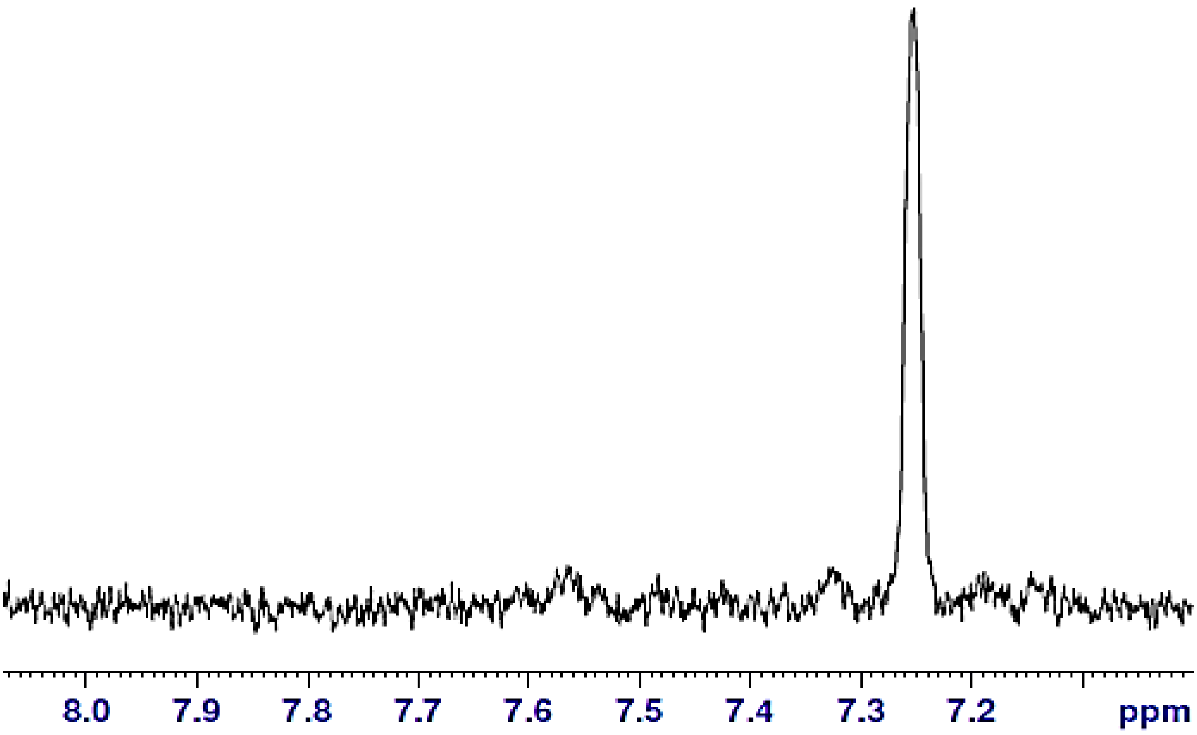

Figure S2. *^{1}H NMR (300MHz, DMSO-D6) spectrum of the reaction products obtained on DFF oxidation catalyzed by the supernatant recovered after the first run with the Sihgel@CTMS@APTMS-GAH@CALB2 biocatalyst. Reaction conditions: 10 mM DFF, 2 mL EtOAc/tBuOH (1:1. v/v), sequential addition of 2.0 equivalents aqueous H_2O_2 (30% v/v) every hour for seven hours, temperature 40 °C, reaction time 24 hours.*

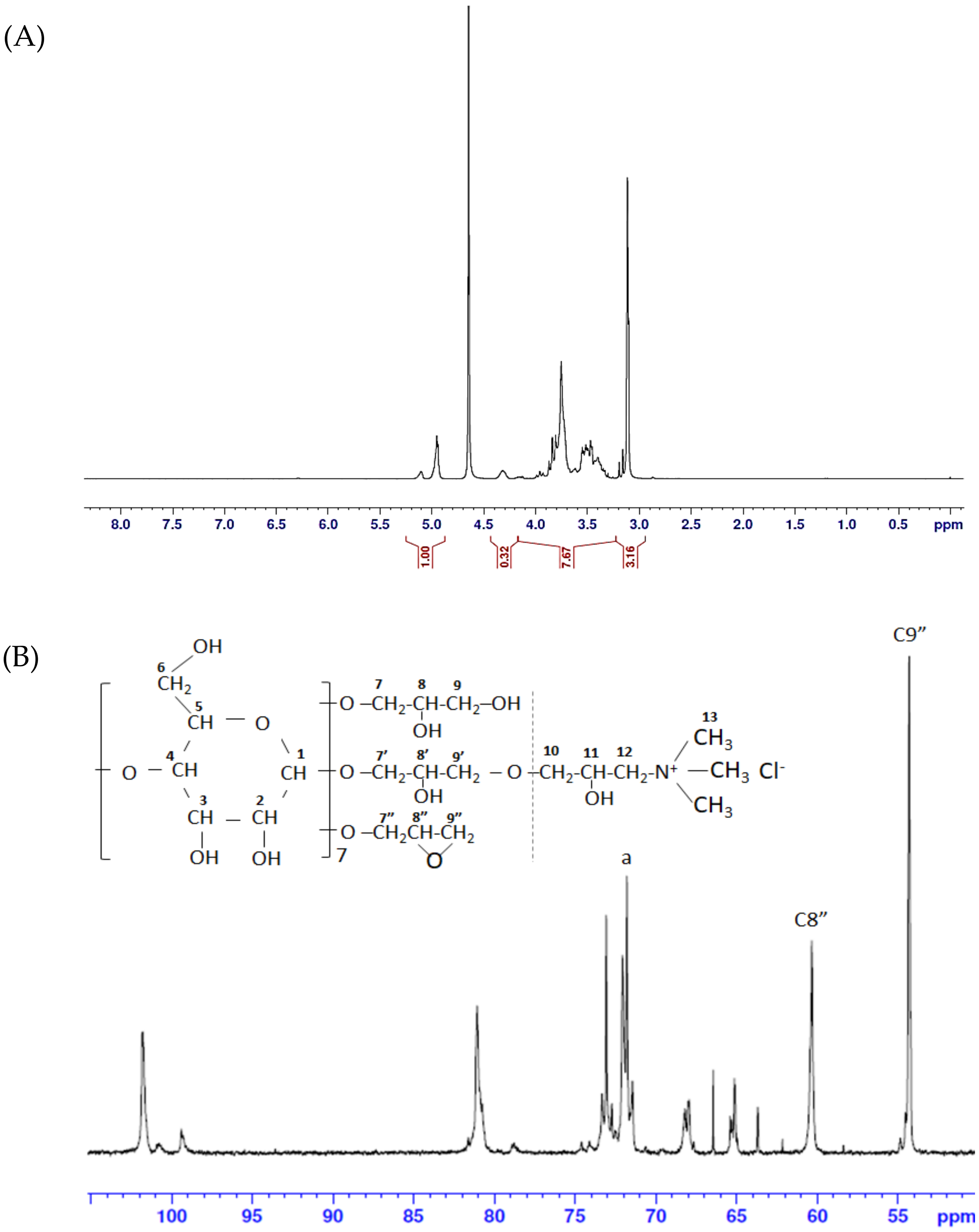

Figure S3. *^{1}H NMR (300MHz, D_2O) (A) and ^{13}C NMR (D_2O, 12000 accumulations) (B) spectra of cationic cross-linked β-cyclodextrin (CCLβ-CD). The signal at 3.16 ppm in (A) is characteristic of the methyl protons from trimethylammonium groups. The resonance a in (B) is typical of C7, C8, C7’, C8’, and C9’ from the 2-hydroxypropyl ether segments [1]. Resonances C8” and C9” indicate also the presence of glycidyl group which may be responsible for gelation of CCLβ-CD after prolonged storage.*

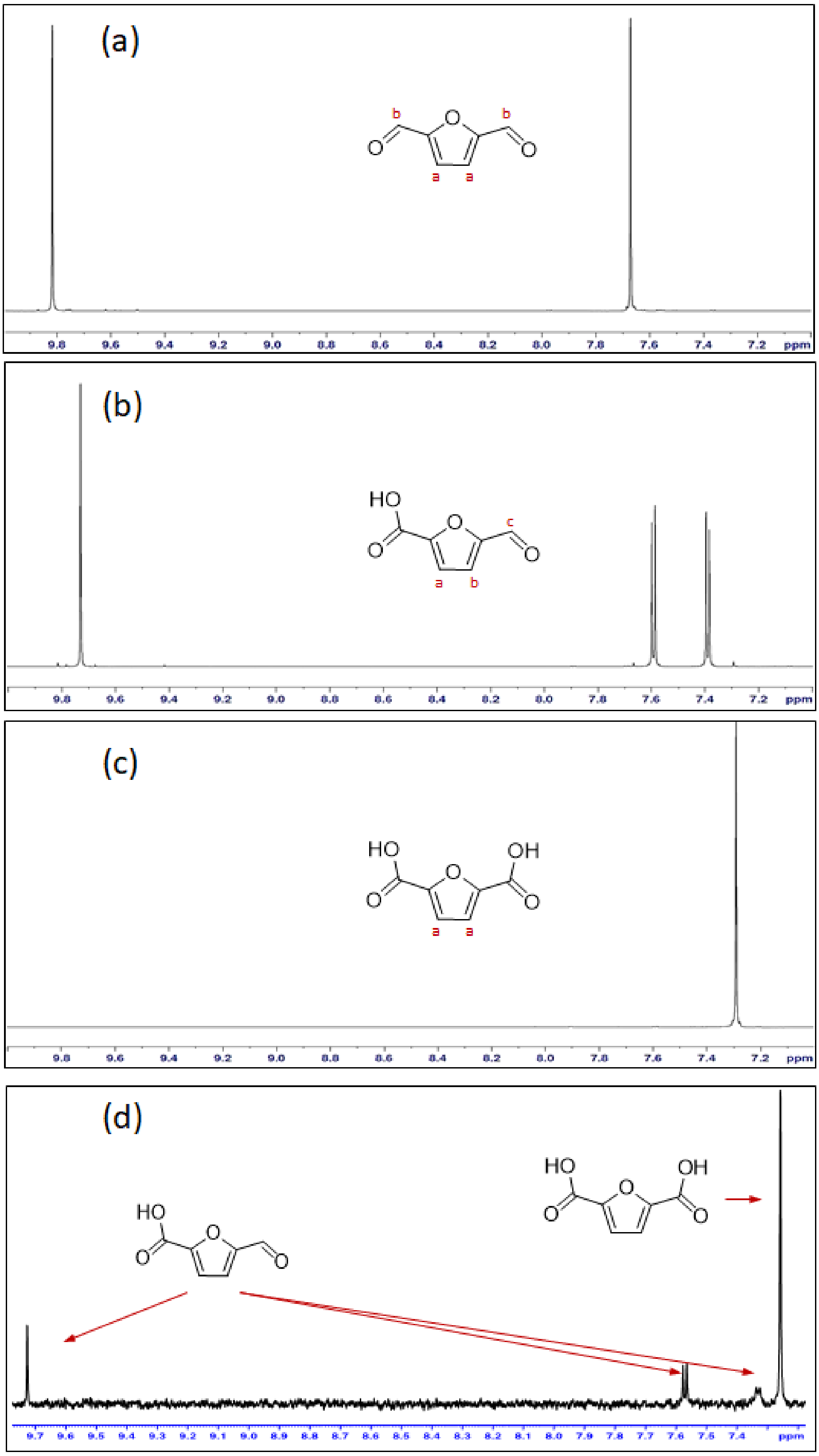

Figure S4. *^{1}H NMR (300MHz, DMSO-D6) spectra of (a) DFF* **a**: *δ = 7.67 (s, 2H, Ar),* **b**: *δ = 9.82 (s, 2H, aldehyde); (b) FFCA* **a**: *δ = 7.39 (d, 1H, Ar),* **b**: *δ = 7.60 (d, 1H, Ar),* **c**: *δ = 9.73 (s, 1H, aldehyde); (c) FDCA* **a**: *δ = 7.30 (s, 2H, Ar); (d) Typical spectrum of reaction products obtained with Sihgel@CTMS@APTMS-GAH@CALB2: DFF conversion 100%; FFCA yield 10% and FDCA yield 90%.*

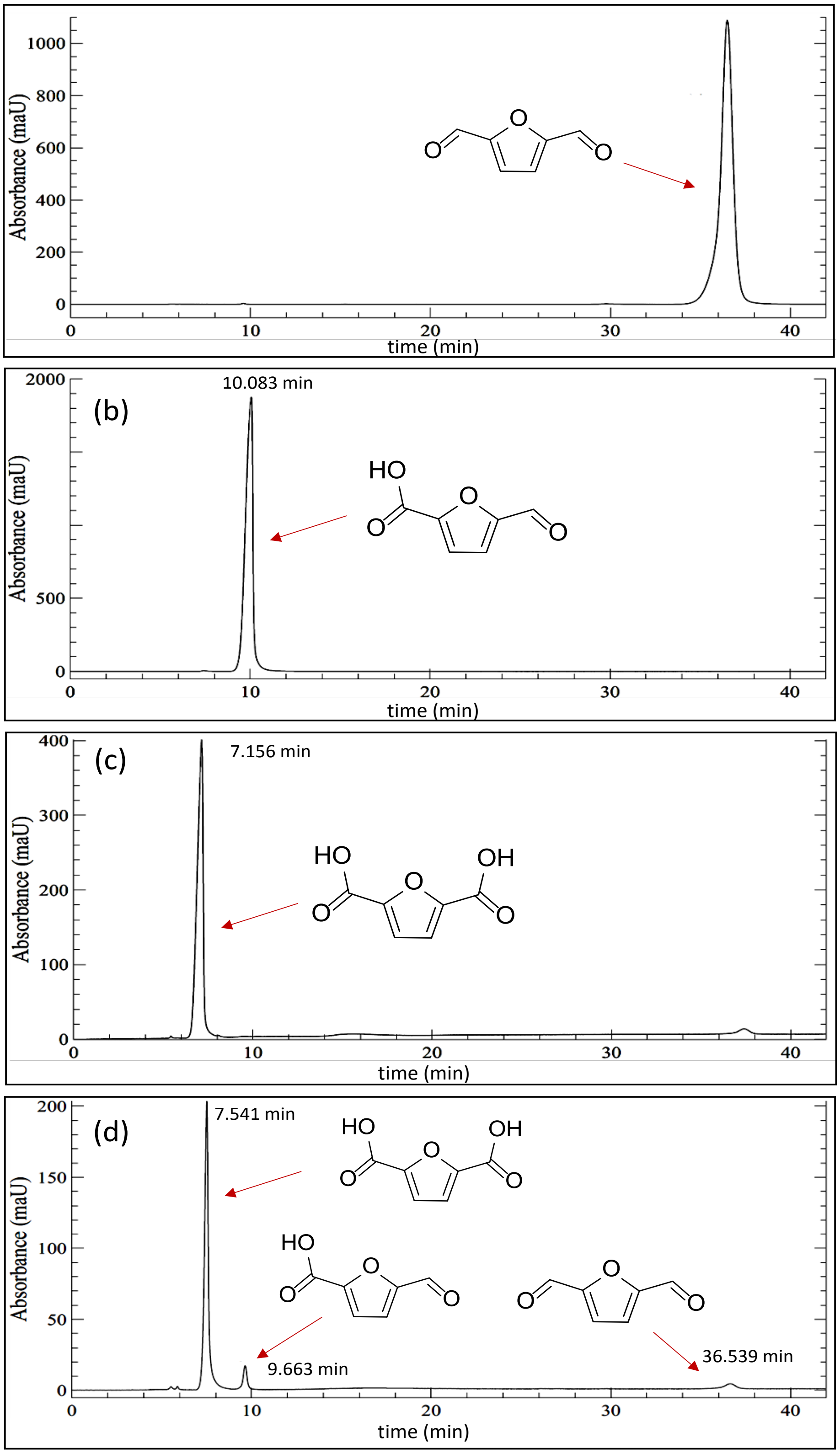

Figure S5. *HPLC chromatograms of standard solutions: (a) DFF; (b) FFCA; (c) FDCA and (d) typical spectrum of the reaction mixture. Analytical conditions: mobile phase acetic acid (0.2%), temperature 60°C, flow rate 0.6 mL/min. UV detection at 284 nm.*

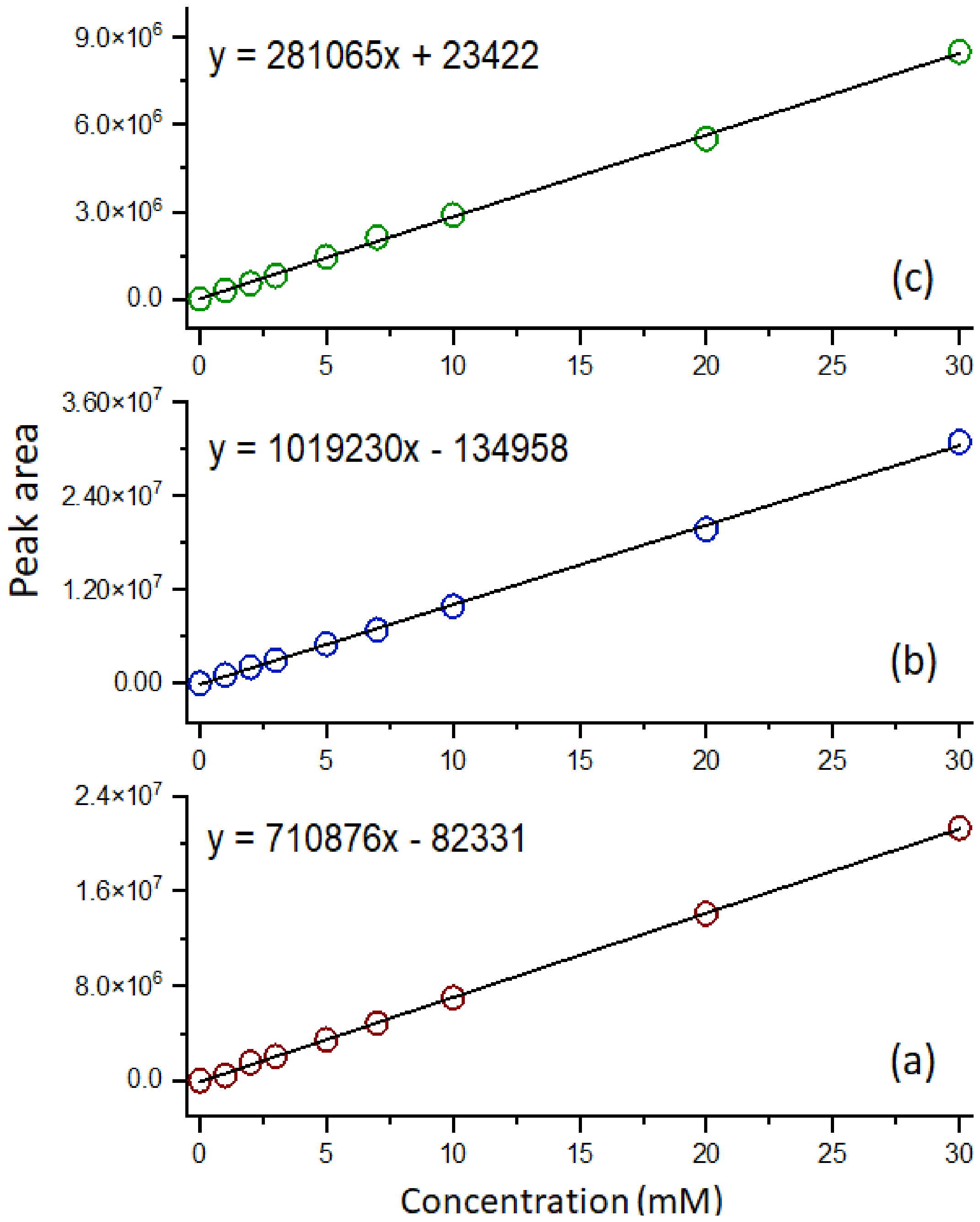

Figure S6. *HPLC calibration curves of (a) DFF, (b) FFCA and (c) FDCA. The analytical conditions were the same as those described in Figure S5.*

---

[i] Renard, E.; Deratani, A.; Volet, G.; Sebille, B. Preparation and characterization of water soluble high molecular weight β-cyclodextrin-epichlorohydrin polymers. *Eur. Polym. J.* **1997**, *33*, 49-57.
